# Differential recruitment of brain circuits during fear extinction in non-stressed compared to stress resilient animals

**DOI:** 10.1038/s41598-023-50830-w

**Published:** 2024-01-25

**Authors:** Jiah Pearson-Leary, Alexander P. Abramenko, Valerie Estela-Pro, Elizabeth Feindt-Scott, Jason Yan, Abigail Vigderman, Sandra Luz, Debra Bangasser, Richard Ross, Leszek Kubin, Seema Bhatnagar

**Affiliations:** 1 Stress Neurobiology Center, Department of Anesthesiology and Critical Care, Children’s Hospital of Philadelphia Research Institute, Philadelphia, PA USA; 2 Department of Biology, Haverford College, Haverford, PA USA; 3 Department of Anesthesiology and Critical Care, The Perelman School of Medicine, University of Pennsylvania, Philadelphia, PA USA; 4 Center for Behavioral Neuroscience, Neuroscience Institute, Georgia State University, Atlanta, GA USA; 5 Department of Psychiatry, Perelman School of Medicine, University of Pennsylvania, Philadelphia, PA USA; 6 Department of Biomedical Sciences, School of Veterinary Medicine, University of Pennsylvania, Philadelphia, PA USA

**Keywords:** Neuroscience, Emotion, Stress and resilience

## Abstract

Dysfunctional fear responses in post-traumatic stress disorder (PTSD) may be partly explained by an inability to effectively extinguish fear responses elicited by trauma-related cues. However, only a subset of individuals exposed to traumatic stress develop PTSD. Therefore, studying fear extinction deficits in animal models of individual differences could help identify neural substrates underlying vulnerability or resilience to the effects of stress. We used a rat model of social defeat in which rats segregate into passively and actively coping rats. In previous work, we showed that passively coping rats exhibit disruptions in social interaction whereas actively coping rats do not display behaviors differently from controls, indicating their resilience. Here, adult male rats exposed to 7 days of social defeat were tested for fear extinction, retention of extinction, and persistence of retention using contextual fear and ethologically-relevant fear tests. Passively coping rats exhibited elevated freezing in response to the previously extinguished context. Analyses of cFos expressing cells across select brain regions showed high correlations within dorsal hippocampal subregions, while passively coping rats had high correlations between the dorsal hippocampus CA1 and the central and basolateral subregions of the amygdala. Importantly, although control and actively coping rats showed similar levels of behavioral extinction, there was little similarity between activated structures, suggesting stress resilience in response to chronic social defeat involves an adaptive differential recruitment of brain circuits to successfully extinguish fear memories.

## Introduction

Post-traumatic stress disorder (PTSD) is a debilitating mental health condition that develops in a subset of people following exposure to traumatic stress^1,2^. PTSD is diagnosed based on clusters of symptoms, including re-experiencing and/or having intrusive memories of trauma, avoidance, negative mood and thoughts, and disruptive levels of hyperarousal^1,2^. Not all individuals exposed to traumatic events develop PTSD, however^1^. Active coping behaviors have been shown to reduce the risk of developing PTSD, while more passive behaviors such as social avoidance can increase the risk of developing PTSD^3^. Using repeated social defeat by an aggressive resident rat, we have shown that resilient and vulnerable subpopulations emerge based on the coping strategies employed during defeat stress^4–11^. Rats passively coping during exposure to an aggressive conspecific exhibit changes in behaviors including reductions in social interaction and increases in immobility during the forced swim test as compared to control animals, indicating their vulnerability to the effects of stress. Actively coping rats display behaviors not different from those of non-stressed controls, indicating their resilience to the effects of stress^5–10^. Thus, studying individual differences in response to repeated social defeat stress could ultimately provide insight into the neural substrates that underlie PTSD and related anxiety disorders.

The dysfunctional fear response that characterizes PTSD may be partly explained by an inability to retain the learned extinction of fear responses to previously traumatic cues^12,13^. This disruption in fear extinction retention can occur both for the traumatic event and for fear memories both related and unrelated to the trauma that induced PTSD^14–17^. Thus, preclinical models in which individual differences in extinction retention are observed would be valuable in identifying how traumatic stress re-organizes brain circuits in subsets of individuals to create extinction-resistant fear memories.

In the current study, we examined fear extinction in rats that exhibited passive or active coping strategies during repeated social defeat. We tested these rats in two different contexts: one in which rats were previously socially-defeated, referred to as ethological fear testing due to its proximity to naturalistic aggression, and another using a standard contextual fear paradigm in which control or socially-defeated rats were exposed to unsignaled foot shocks that produce context-specific conditioned fear^18^. Next, we examined functional connectivity via correlations of numbers of c-Fos-expressing cells between brain regions known to be important in stress, fear, and memory following extinction retention testing to identify putative circuits that may underlie vulnerability vs. resilience to the effects of stress on fear learning. Our findings demonstrate that stress vulnerable/passively coping rats show impairments in the retention of fear extinction. Active coping/resilient rats show successful retention of fear extinction similar to controls, however, the response in active coping rats is likely through an adaptive switch in brain circuits recruited during extinction training.

## Results

### Experiment 1: ethological fear testing

**Figure 1:**
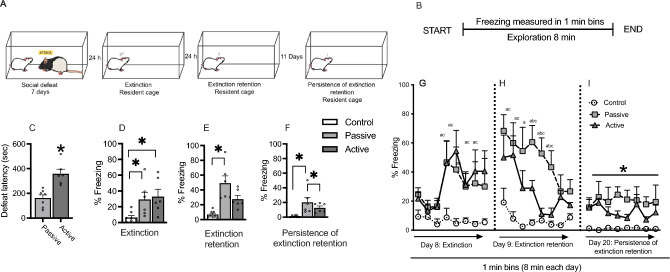
Fear extinction in response to environment where rats were previously socially-defeated. (**A**) Experimental design for the testing paradigm. (**B**) Extinction testing protocol to assess freezing when the rat was placed in an empty-resident cage where social defeat had previously occurred 24 h, 48 h or 11 days earlier. (**C**) Latencies to social defeat showing splits expressed by animals tested in this experiment. (**D**–**F**). Bar graphs showing percent total freezing over the course of the 8 min testing trial over the 3 testing days and results of Tukey’s post hoc tests on significant one-way ANOVAs. (**G**–**I**), Repeated measures analyses of freezing responses separated into 1 min bins, with time as the repeated measure. At days 8 and 9, there were interaction effects, with post hoc tests revealing differences between passive coping, active coping, and/or control rats at several time points. I. On day 20, there was a main effect of group, as shown by an asterisk indicating that passive coping rats had high freezing relative to active coping and control rats. Post hoc significance symbols: Passive vs control = a, passive vs active = b, active vs non-stressed control = c. * *p* < 0.05 for main effect shown in I. Control n = 8, passive n = 7, active n = 6.

Rats were socially defeated for 7 days (Fig. 1A). During each episode of social stress, a rat was placed into the home cage territory of an unfamiliar Long-Evans resident previously screened for high aggression. A typical agonistic encounter resulted in intruder subordination or defeat, signaled by the intruder assuming a supine position for 3 s. After defeat, a wire mesh partition was placed in the cage to prevent physical contact between the resident and intruder but allowing visual, auditory, and olfactory contact for the remainder of the 30 min defeat session. On each day, intruder rats were placed in the home cage of a different resident aggressor for 15 min or until the intruder displayed a supine and frozen defeat posture, whichever occurred first. This time to show the defeat posture was recorded as latency to be defeated. One set of resident rats was used for each cohort of intruder rats. Thus, all intruder rats in a given cohort were exposed to the same set of residents but on a different day. This minimized the impact of variations in aggression across the cohort of resident rats. Daily latencies to be defeated displayed by intruder rats were averaged across the 7 days. If an intruder resisted defeat for 15 min, the resident and intruder were separated with the wire partition for the remainder of the session. Controls were placed behind a wire partition in a novel cage for 30 min daily. Rats were returned to their home cage after each session. To identify passively or actively coping rats, the average latency of each rat over the course of 7 days of defeat was entered into an R script used to perform bootstrap cluster analysis (code available at www.github.com/cookpa/socialdefeat). The analysis provides probabilities for resilience, with 1 indicating resilience and 0 indicating vulnerability, with 0.5 being the point of delineation between actively and passively coping rats. Latencies clustered at 161 s ± 26.3 s (N = 7) for passive coping rats and 359 s + / − 37.1 s (N = 6) for active coping rats (*t* = 4.44, *p* = 0.001; Fig. 1C). The latencies for each group are similar to our previous results^5–8^. To measure fear expression and extinction in an ethologically relevant way, rats were then re-exposed to the cage of the resident by which they were last defeated without the resident in the cage as extinction training (day 8), extinction retention testing (day 9), and persistence of extinction retention (day 20) (Fig. 1A, B).

#### Experiment 1.1. Both passive coping and active coping rats display higher levels of ethological fear compared to novel cage control rats

Both passively and actively coping stress-exposed rats displayed a higher percentage of time spent freezing during extinction (F_2,17_ = 4.8, *p* = 0.02, Fig. 1D) as compared to rats that were not exposed to social defeat stress and were instead placed in a novel cage daily for the duration of the social defeat paradigm (novel cage controls). In addition, a repeated measures ANOVA revealed a main effect of time (F_7,119_ = 8.85, *p* = 0.0001) and stress group (F_2,17_ = 4.86, *p* = 0.02) and a significant interaction effect (F_14,119_ = 2.43, *p* = 0.005; Fig. 1G). The post-hoc tests indicated that passive coping and active coping rats both exhibited increased freezing relative to non-stressed control rats at multiple timepoints (Fig. 1D, G) during the extinction training phase.

#### Experiment 1.2. Passive coping rats show reduced retention of extinction and this reduction persists over time

Following extinction training (day 8), we tested freezing in response to the aggressor’s cage on day 9 (extinction retention) and day 20 (persistence of extinction retention) without the aggressor resident rat present. On day 9 during extinction retention testing (Fig. 1E, H), a two-way repeated measures ANOVA revealed main effects of time (F_7,119_ = 8.87, *p* = 0.0001) and group (F_2,17_ = 12.51, *p* = 0.005), and a significant interaction effect (F_14,119_ = 2.14, *p* = 0.001) were observed). Post-hoc analysis of the main group effect revealed that passive coping rats showed increased freezing compared to non-stressed control rats (Fig. 1E). Further, post-hoc analyses for differences at individual time points confirmed that passive coping rats had increased freezing relative to active coping rats at time points 4, 5, and 6 (denoted by letters) and increased freezing relative to non-stressed control rats at time points 1–6 (Fig. 1H). Active coping rats had increased freezing relative to control rats only at time point 2. When examining freezing behaviors during testing for persistence of extinction retention (day 21; Fig. 1F, I), a two-way repeated measures ANOVA revealed a main effect of group (F_2,17_ = 7.715, *p* = 0.006). Post-hoc analyses revealed that passive coping rats had increased freezing relative to both active coping and control rats. These data suggest that extinction retention deficits persist over time in passive coping rats when tested for fear responses in an environment where social defeat previously occurred.

### Experiment 2: Standard contextual fear testing

**Figure 2:**
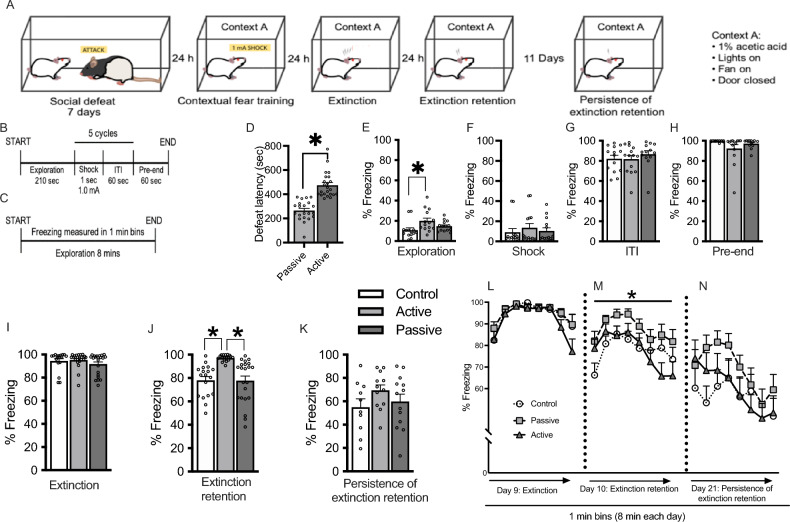
Contextual fear following social defeat stress. (**A**) Experimental design for the testing paradigm utilizing 7 days of social defeat and 3 days of contextual fear testing. (**B**–**C**). Protocol for contextual fear training (**B**) and extinction and extinction retention testing (**C**), leading to robust fear expression in response to the contextual cue. (**D**) Latencies to social defeat showing splits exhibited by animals tested in this experiment. (**E**–**H**). Total freezing during conditioning averaged individually across the exploration, shock, ITI, or pre-end portion of the training protocol and assessed by one-way ANOVA followed by Tukey post-hoc tests. (**I**–**K**). Total freezing expressed as a percent of time spent freezing in 1 min bins over the 8 min testing session for extinction (I, day 9), extinction retention (J, day 10), and persistence of extinction retention (K, day 21). (**L**–**N**), Repeated measures analyses of freezing responses separated into 1 min bins, with time as the repeated measure. Performance on each day was tested by a two-way repeated measures ANOVA using time and stress group (control, passive coping, and active coping) as factors). *p < 0.05. Control n = 18, passive coping n = 20, active coping n = 22.

Similar to Experiment 1, rats were socially defeated for 7 days. Here, following social defeat we trained rats in a standard contextual fear paradigm (Fig. 2A) based on Knox et al.^19^. Seven days of social defeat led to a split in defeat latencies with passive coping rats having an average of 265 s ± 17.9 s (N = 21) to social defeat, and active coping rats showing an average of 475 s ± 21.6 s (N = 23) to defeat (*t* = 7.38, *p* < 0.0001; Fig. 2D). These latencies are in the range of our previous results^5–8^.

#### Experiment 2.1. Increased baseline freezing in passive coping rats

Data were averaged during each period of contextual fear training on day 8, and freezing was subsequently assessed during exploration (210 s total), shock (5 s total), the inter-trial interval (ITI; 300 s total), and pre-end (60 s total; see timeline in Fig. 2B). Data were analyzed within each training segment specifically to assess whether there were differences in the amount of freezing that occurred prior to delivery of shocks that persisted past training. Passive coping rats had increased freezing relative to control rats during the exploration phase of training (F_2,39_ = 4.169, *p* = 0.02, Fig. 2E). There were no differences in other measures (Fig. 2F,G,H). By the end of the training trial (pre-end period), all rats displayed close to maximum levels of freezing (Fig. 2H), indicating they had successfully associated the context with fear.

#### Experiment 2.2. Passive coping rats have reduced extinction retention relative to active coping and control rats

We then examined freezing in the contextual fear chamber during extinction training (day 9), extinction retention (24 h later, day 10), and persistence of extinction retention (11 days following extinction retention testing, day 21). A two-way repeated measures ANOVA showed no differences in freezing during the extinction training (day 9, Fig. 2I, L). All rats displayed high levels of freezing during this phase, indicating strong contextually conditioned fear behavior. A two-way repeated measures ANOVA on freezing during extinction retention (day 10, Fig. 2J, M) revealed significant main effects of time (F_7,224_ = 5.84, *p* = 0.001) and group (F_2,32_ = 5.281, *p* = 0.01). There was no interaction effect. Post-hoc tests revealed that passive coping rats had significantly increased freezing relative to non-stressed control rats, while active coping and non-stressed control rats did not differ from each other. These results indicate that passive coping rats had impaired extinction retention relative to active coping rats and non-stressed control rats, but did not show deficits in persistence of extinction retention in the contextual fear paradigm (day 21, Fig. 2K, N) in the manner they did in the ethological fear paradigm in Experiment 1.

### Experiment 3: Analysis of neuronal activity and inter-regional correlations

**Figure 3:**
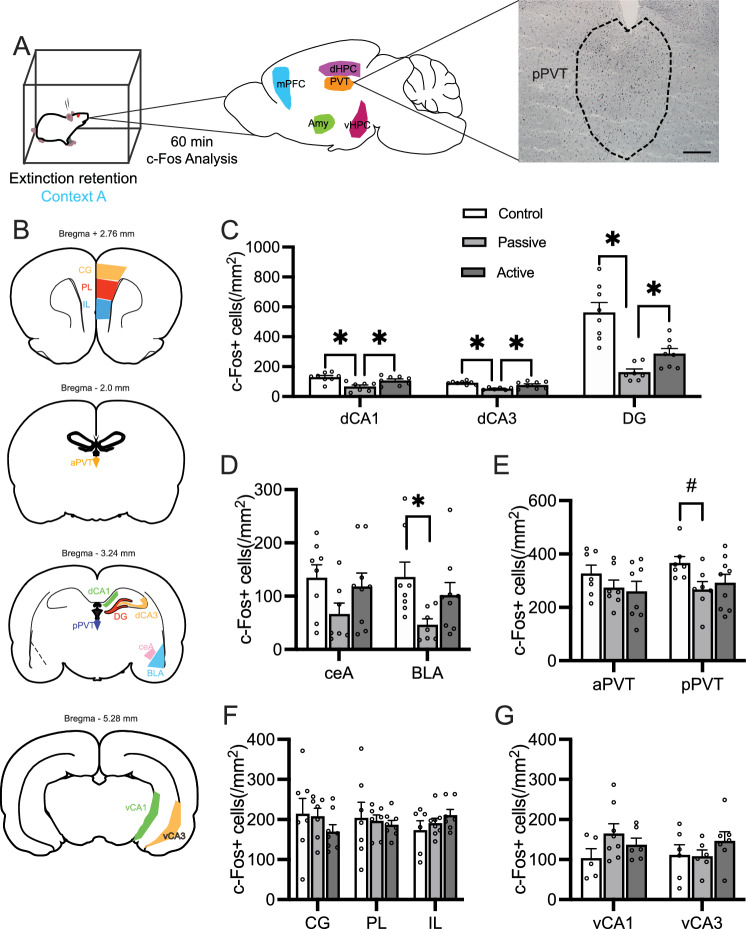
Analysis of c-Fos in selected brain regions following extinction retention testing. (**A**) Experimental overview of brain regions examined by c-Fos immunohistochemistry following extinction retention testing. (**B**) Regions of interest analyzed within the brains of each subject. Passive coping rats had reduced c-Fos in (**C**) dCA1, dCA3, and DG as well as in (**D**) BLA, although not in ceA. No changes in c-Fos expression were seen in the aPVT, but there was a trend toward lower expression by passive coping animals in pPVT. No differences were observed in the mPFC (**F**) or vHPC (**G**). Data analyzed by one-way ANOVA followed by Tukey’s post-hoc tests on significant results, *p < 0.05, # *p* < 0.1. CG, anterior cingulate cortex; PL, prelimbic cortex; IL, infralimbic cortex; aPVT, anterior paraventricular thalamus; pPVT, posterior paraventricular thalamus; ceA, central amygdala; BLA, basolateral amygdala; dCA1, dorsal cornu Ammonis-1; dCA3, dorsal cornu Ammonis-3; vCA1, ventral cornu Ammonis-1; vCA3, dorsal cornu Ammonis-3. Scale bar = 200 μm. Control n = 8, passive coping n = 7, active coping n = 9.

Following extinction retention testing (day 10), a subset of rats trained in the contextual fear paradigm were analyzed for numbers of c-Fos-expressing cells and inter-region correlations of c-Fos-expressing cells. The density of c-Fos staining in a given region is widely used as a marker for neuronal activation, and inter-region correlations of c-Fos densities may suggest functional connectivity and/or highlight important differences in regionally specific activity between animals with differing levels of oping. Following extinction retention testing (day 10), we examined the density of c-Fos-expressing cells (c-Fos positive cells/total cells) in selected brain regions known to be involved in contextual fear, extinction, and memory (Fig. 3A, B). We assessed cFos on day 10 as freezing on this day was most strongly differentiated between passively and actively coping rats and control rats.

#### Experiment 3.1. Passive coping rats have reduced neuronal activity in the dorsal hippocampus and amygdala during extinction retention testing

Passively coping rats showed lower neuronal activation in hippocampal regions, the BLA, and the posterior PVT compared to actively coping and control rats. Specifically, there was significantly lower activity in dorsal CA1 (dCA1; F_2,20_ = 8.29, *p* = 0.002), dorsal CA3 (dCA3; F_2,19_ = 9.627, *p* = 0.05) and in dentate gyrus (DG; F_2,20_ = 19.98, *p* = 0.0001; Fig. 3C) of passive coping rats relative to active coping and control rats (Fig. 3C). Passive coping rats had reduced numbers of c-Fos positive cells in the basolateral amygdala (BLA; F_2,21_ = 3.536, *p* = 0.04; Fig. 3D), but no significant differences in the central amygdala (ceA). There were no group differences in c-Fos expression in the anterior paraventricular nucleus of the thalamus (aPVT; Fig. 3E), medial prefrontal cortex (prelimbic, PL; infralimbic, IL), cingulate (CG; Fig. 3F), or ventral hippocampus CA1 (vCA1) or CA3 (vCA3) regions (Fig. 3G). There was a trend toward lower c-Fos expression in the posterior paraventricular nucleus of the thalamus (pPVT, F_2,20_ = 2.77, *p* = 0.08). Correlations performed between numbers of c-Fos-expressing cells and percent freezing indicated no significant association between neural activity in any brain region and freezing behavior during extinction retention.

#### Experiment 3.2. Differences in inter-region correlations of c-Fos within passive coping, active coping, and control populations following extinction retention testing

**Figure 4:**
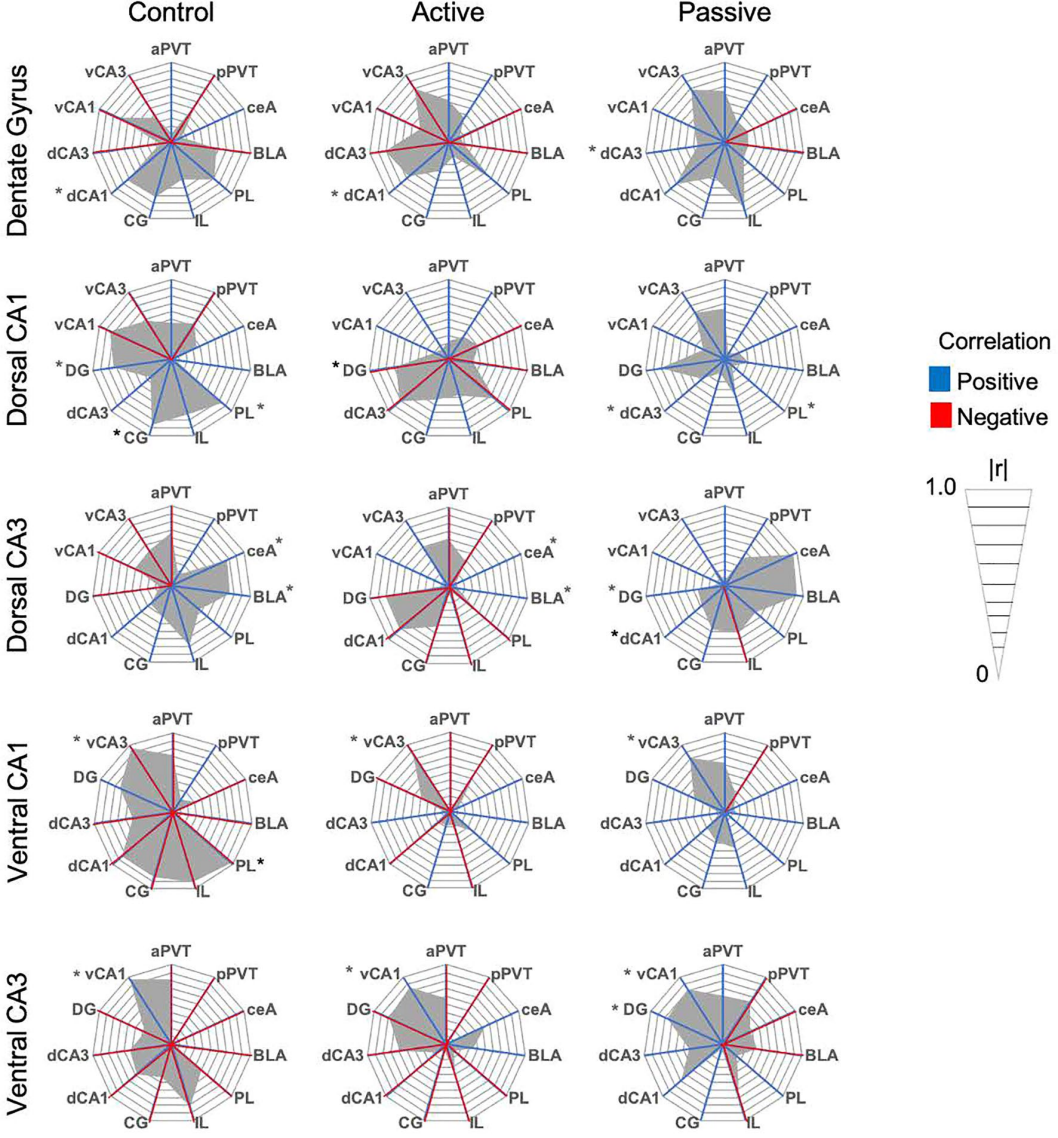
Radar graphs made per brain region demonstrating correlations between regions for the dorsal and ventral hippocampus and dentate gyrus. Red lines indicate negative correlations, while blue lines indicate positive correlations. The gray region represents the degree of correlation on an absolute scale presented in *r* = 0.1 intervals. *p < 0.05.

**Figure 5:**
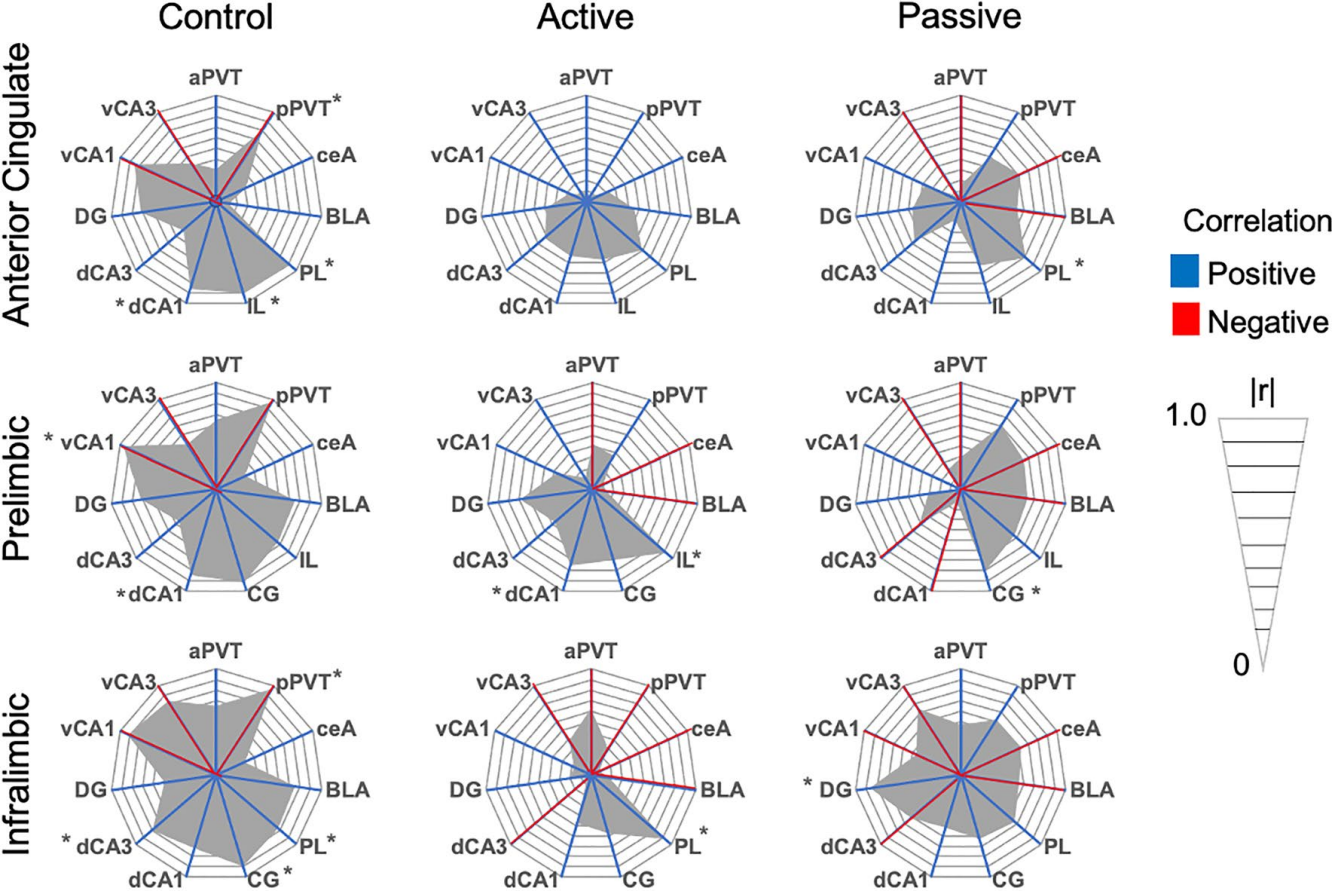
Radar graphs made per brain region demonstrating correlations between regions for the medial prefrontal cortex. Red lines indicate negative correlations, while blue lines indicate positive correlations. The gray region represents the degree of correlation on an absolute scale presented in *r* = 0.1 intervals. *p < 0.05.

**Figure 6:**
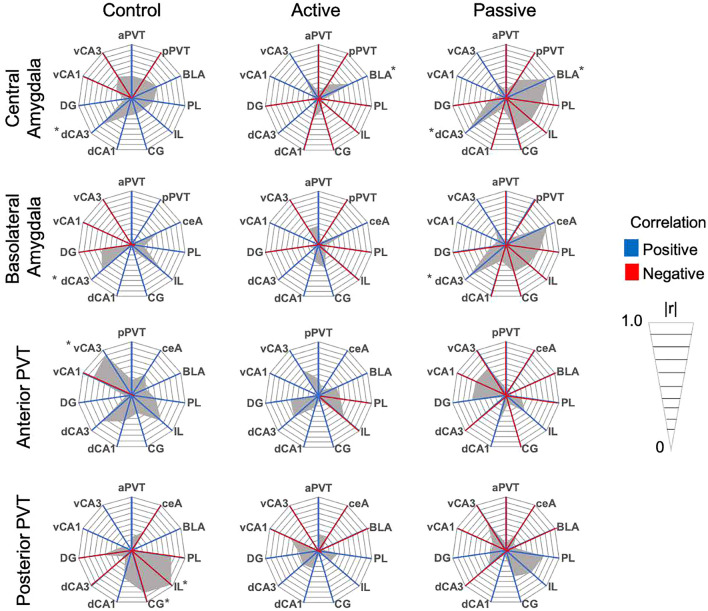
Radar graphs made per brain region demonstrating correlations between regions for the amygdala and PVT. Red lines indicate negative correlations, while blue lines indicate positive correlations. The gray region represents the degree of correlation on an absolute scale presented in *r* = 0.1 intervals. *p < 0.05.

To assess functional connectivity between brain regions, we first assessed inter-region c-Fos correlations within-subjects (Table 1; Radar graphs of all within-subject correlations are presented in Figs. 4, 5, 6). Control rats had 13 significant correlations, while passive coping and active coping rats had 6 each (see Table 1). This difference reveals that net levels of functional connectivity appear to be reduced by stress exposure. Of the significant correlations, passive coping and control rats shared 4 significant correlations, while passive coping and active coping rats shared 2 significant correlations. The only regions that showed significant inter-region correlation in all three groups was the ventral hippocampus (vCA1 vs. vCA3). The only shared significant correlation between control and active coping rats was the positive correlation seen between the PL and dCA1, while passive coping rats did not show such correlated activity between these regions. This could suggest that high functional connectivity between the PL and dCA1 is important for successful fear extinction retention. Control rats had negative correlations between the aPVT and vCA3, pPVT and IL, and pPVT and CG. Neither active coping nor passive coping rats showed any significant correlations with the PVT and other regions. This suggests that a history of stress might reduce the correlation of activity of the PVT with other regions during fear extinction retention testing. Overall, there were greater levels of inter-region correlations in control rats, suggesting that social defeat stress had a broad effect on reducing correlative c-Fos activity across the regions studied, and this could indicate reduced communication between these regions.

**Table 1:** Within-subject Pearson’s *r* correlations of c-Fos immunoreactivity between selected brain regions. *p < 0.05. Significant values are in bold.

	Correlations		
	Control	Passive coping	Active coping
	**r**	**r**	**r**
aPVT vs vCA3	r = − 0.81	r = − 0.06	r = 0.57
	*p* = 0.049*	*p* = 0.907	*p* = 0.113
pPVT vs IL	r = − 0.97	r = 0.62	r = 0.29
	*p* = 0.005*	*p* = 0.186	*p* = 0.518
pPVT vs CG	r = − 0.81	r = 0.50	r = 0.06
	*p* = 0.048*	*p* = 0.303	*p* = 0.893
ceA vs BLA	r = 0.52	r = 0.85	r = 0.66
	p = 0.356	*p* = 0.015*	*p* = 0.05*
ceA vs dCA3	r = 0.76	r = 0.95	r = 0.09
	*p* = 0.027*	*p* = 0.003*	*p* = 0.828
BLA vs dCA3	r = 0.74	r = 0.91	r = 0.11
	*p* = 0.035*	*p* = 0.01*	*p* = 0.795
PL vs IL	r = 0.76	r = 0.67	r = 0.91
	*p* = 0.075	*p* = 0.142	*p* = 0.001*
PL vs CG	r = 0.91	r = 0.80	r = 0.69
	*p* = 0.004*	*p* = 0.053	*p* = 0.055
PL vs dCA1	r = 0.84	r = − 0.12	r = 0.75
	*p* = 0.017*	*p* = 0.81	*p* = 0.049*
PL vs vCA1 IL vs CG	r = − 0.97	r = 0.01	r = 0.37
	*p* = 0.029*	*p* = 0.976	*p* = 0.469
	r = 0.90	r = 0.63	r = 0.57
	*p* = 0.014*	*p* = 0.18	*p* = 0.139
IL vs dCA3	r = 0.79	r = − 0.62	r = − 0.05
	*p* = 0.050*	*p* = 0.262	*p* = 0.915
IL vs DG	r = 0.48	r = 0.87	r = 0.19
	*p* = 0.326	*p* = 0.024*	*p* = 0.67
CG vs dCA1	r = 0.86	r = 0.18	r = 0.53
	*p* = 0.012*	*p* = 0.728	*p* = 0.22
dCA1 vs dCA3	r = 0.35	r = − 0.38	r = 0.81
	*p* = 0.391	*p* = 0.454	*p* = 0.014*
dCA1 vs DG	r = 0.73	r = 0.82	r = 0.67
	*p* = 0.037*	*p* = 0.023*	*p* = 0.068
dCa3 vs DG	r = − 0.15	r = − 0.34	r = 0.80
	*p* = 0.723	*p* = 0.501	*p* = 0.015*
vCA1 vs vCA3	r = 0.96	r = 0.81	r = 0.85
	*p* = 0.032*	*p* = 0.047*	*p* = 0.029*

#### Experiment 3.3. Differences in c-Fos correlations between passive coping, active coping, and control rats following extinction testing

To statistically compare correlations in c-Fos densities across pairs of regions between control, passive coping, and active coping populations, we converted Pearson’s *r* values to *z*-scores using Fisher’s *r* to *z* transformation, which normally-distributes *r* values and allows statistical comparisons between groups using the *z*-test (Table 2). In the control vs. passive coping comparison, there were 5 significantly different correlation comparisons. In the control vs. active coping comparison, there were 4 significantly different comparisons. There were 5 significantly different comparisons between active coping and passive coping rats. The negative correlation between the pPVT and the IL was significantly different between control rats and both passive coping and active coping rats. This suggests that prior stress exposure prevents the reductions in functional connectivity between the pPVT and IL that occurs in subjects without a prior history of social defeat stress. The significant differences in c-Fos correlations between passive coping and active coping rats all occurred between the amygdala and dorsal hippocampus, and within dorsal hippocampus subregions. Notably, active coping rats, unlike both control and passive coping rats, showed strong correlations between the dCA3 and DG. This suggests that internal circuitry within the dorsal hippocampus during fear extinction retention testing could be an important element of resiliency. An important take away from these results is that while control and active coping rats had similar performance on the contextual fear test, different brain regions were activated during these tests. These results suggests that active coping rats show circuit-based adaptations to the effects of repeated stress that prevent extinction deficits.

**Table 2:**
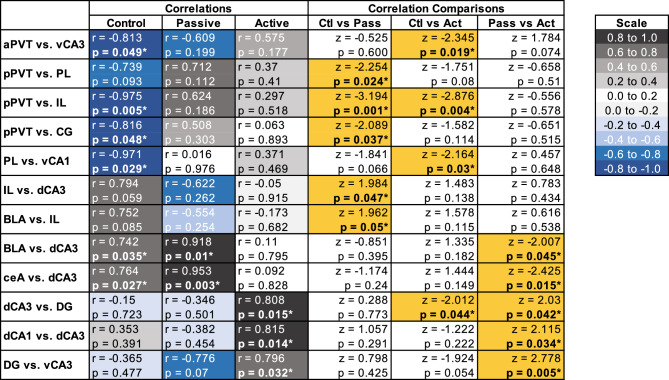
Significant between-subjects comparisons of Pearson’s *r* correlations tested by Fisher’s *z*-tests. Significant correlation comparisons are highlighted in yellow.

## Discussion

The overall goal of this study was to characterize the temporal dynamics of fear extinction and the retention of extinction in stress resilient and vulnerable subpopulations of rats using two separate conditioned fear paradigms: one a naturalistic paradigm employing cues from the cage of a resident aggressor in which the subject was previously defeated in order to evoke fear memories, and the other a standard contextual fear paradigm that uses contextual cues to evoke fear memories. The focus on retention of extinction was informed by both previous studies^19^ as well as findings from PTSD patients themselves: individuals with PTSD not only have reduced ability to successfully extinguish fear memory in response to trauma-related cues, but also a reduced ability to successfully reduce fear responses to memories created within experimental settings^20^. Our results reveal that passively coping rats have impaired extinction retention in both paradigms tested, suggesting that they informative for our understanding of PTSD-like symptoms.

In Experiment 1, both passive coping and active coping rats showed increased freezing during extinction training when placed in the cage of the resident that had last defeated them. However, while active coping rats showed reduced freezing 24 h following extinction training (during extinction retention testing), passive coping rats failed to reduce their freezing. This failure to extinguish the fear-associated memory persisted long-term in this ethologically relevant paradigm, as evidenced by increased freezing when tested again within the previously encountered aggressor’s cage 11 days later. Passively coping and actively coping rats were not significantly from each other in extinction retention but passively coping rats exhibited elevated freezing when tested 11 days later showing their impairment in extinction retention persisted. These findings suggest that the underlying circuitry in passive coping rats may prevent the successful retention of a fear extinction memory. In contrast, the circuitry in actively coping rats may underlie why extinction is persistent in some individuals.

In Experiment 2, in which a standard contextual fear conditioning paradigm was used following chronic social defeat, results were largely similar to those in Experiment 1. All groups showed high freezing when tested in the extinction training trial (day 9), perhaps constituting a ceiling effect. However, when tested for extinction retention on day 10, passive coping rats showed increased freezing relative to control rats, but actively coping rats were not different from controls. While we have interpreted this as a failure of these passively coping rats to retain memory for extinction, reductions in freezing across trials of extinction retention during both experiments may instead indicate impairment of extinction re-learning. While the results indicate that actively coping rats are exhibiting reduced freezing during fear learning, it is possible that actively coping rats are displaying passive behaviors that were not captured by our assessments of latencies to be defeated, such as darting or risk assessment. More detailed analyses of behaviors during defeat would be helpful to more fully understand the behavioral strategies used by active and passively coping rats.

In order to determine the neural circuits that could underlie the reduced retention of extinction in passive coping rats, we examined correlations in neuronal activity between regions involved in contextual fear acquisition and extinction following extinction retention testing in the shock-induced contextual fear paradigm as the specific circuits employed in this paradigm have been well-validated^16,21–28^. Results of the c-Fos analyses showed a reduction in activity in the dorsal hippocampus and BLA of passive coping rats relative to control and active coping rats. This reduction in numbers of c-Fos-expressing cells in the BLA was found in the BLA of passive coping rats, despite these animals showing higher freezing. Traditionally, BLA activation is associated with high freezing, however, this relationship may depend on the specific source of inputs to the BLA^29,30^. This suggests that these regions are important for the expression of extinction responses (i.e., less freezing in an extinguished fear-eliciting context) that were observed, and that passive coping rats fail to activate emotionally-salient contextual information important for guiding the expression of extinction. By examining c-Fos profiles and correlations across relevant brain regions, we were then able to infer potential functional connectivity between regions. Since the neural circuits that are important for long-term retention of extinction memories are not well-established, our selection of regions for study was based on fear conditioning-, extinction-, and stress-relevant circuits. Intriguingly, despite low levels of c-Fos activity in the dorsal hippocampus and amygdala, passive coping rats showed high functional connectivity between the dCA3 and both the BLA and CeA. While control and active coping rats had similar levels of freezing during extinction retention testing, they showed marked differences in functional connectivity between the brain regions we assessed. Active coping rats had high levels of connectivity within the dHPC, potentially suggesting a robust activation of contextual representations of the extinction memory, which could drive the display of reduced freezing.

Control rats had more than twice the number of significant inter-region correlations as passive coping or active coping rats. This could indicate that other regions not measured but implicated in previous studies of fear extinction, such as the nucleus accumbens or striatum^31,32^ might be recruited in passive coping and/or active coping rats to regulate fear expression during extinction retention testing. The only correlation common to both control and active coping rats was the strong positive correlation between the PL and dCA1, which was not observed in passive coping rats. These data suggest that dCA1, which sends projections to the PL through intermediate and/or direct connections^28,33,34^, could be an important component of the circuit for accessing fear extinction memory.

We examined c-Fos across the anterior–posterior axis of the PVT as neuronal activity in the PVT regulates habituation to repeated stress and responses to novel stress in chronically stressed rats^35–39^. One interpretation of reduced freezing following several days of exposure to a fear-evoking stimulus could be explained by habituation, rather than extinction. Neither the anterior nor posterior portion of the PVT showed significant differences in c-Fos staining across groups, although there was a trend toward lower c-Fos expression in the pPVT (as seen in Fig. 3E). Collectively, these data suggest that differences in freezing were related to extinction-related processes, rather than habituation-related processes. Intriguingly, correlative activity between the PVT and the mPFC differentiated defeated rats from non-defeated control rats suggesting that a prior history of stress alters the engagement of PVT-related circuits in both passive coping and active coping rats.

Successful fear extinction retention involves both acquisition of a fear extinction memory and its retrieval or recall^40^. While there were no obvious differences during extinction acquisition, we could not definitively determine if there was a deficit in the acquisition of fear extinction or in retrieval during retention testing. Results from our functional connectivity analyses, however, could inform future studies aiming to parse out the role of identified circuits in regulating these distinct components of fear extinction retention. The circuits regulating fear extinction and its retention in stressed females are not clear as our study only examined male animals. It is possible that different brain structures are engaged during fear extinction in females and/or the functional connectivity between the same structures identified here is different in females. Taken together, results from this study demonstrate that successful fear extinction in stress resilient rats involves recruitment of new brain circuits during fear extinction retention testing. Indeed, one of the most intriguing findings was that while the behavior of both control and active coping rats is similar, the underlying circuitry recruited to mediate those behaviors is different. These findings suggest that resilience to the effects of stress is produced by recruitment of specific and unique neural circuitry, not just greater or lesser recruitment of circuitry activated in vulnerable individuals or in non-stressed individuals. Importantly, these unique circuits provide a novel target for promoting resiliency.

## Methods

All experimental procedures were carried out with the approval of the Institutional Animal Care and Use Committee of The Children’s Hospital of Philadelphia Research Institute and in accordance with the NIH guidelines for the care and use of laboratory animals (National Institutes of Health Publication No. 80–23, revised 1996). The methods of the present study have been reported in accordance with ARRIVE guidelines.

### Animals

Adult male Sprague–Dawley rats (225–250 g) were obtained from Charles River Laboratories. Rats were singly housed under a 12-h light–dark cycle (lights on at 7 am and off at 7 pm) and were given food and water ad libitum. All rats were randomly assigned to groups by a lab member that was not involved with experimental procedures or data analyses. Rats were euthanized by rapid decapitation and their brains were immediately snap-frozen in 2-methylbutane in Experiments 2 and 3.

### Experiment 1: ethological fear testing

To assess extinction of fear responses to social defeat, control and socially-defeated rats (passive coping or active coping) were videotaped on day 8 during an 8-min exposure to the empty cage of the resident rat that had defeated them the previous day. On day 7, rats were tested again for freezing in response to the same resident’s cage on day 9 to test for extinction retention and day 20 to test for persistence of extinction retention. Residents remained in their cages in between sessions, and were removed during fear extinction testing to ensure olfactory cues (urine, feces, dander, pheromones, etc.) were available to subject rats. Control rats were exposed to the un-inhabited empty cage they were previously placed in. The experimental design is depicted in Fig. 1A and B. A trained experimenter blind to group condition measured freezing behavior in the videos. Freezing was defined as the absence of movement, except movement necessary for breathing, for greater than 2 s, and quantified as percentage of total time for each session. The total number of subjects used in this experiment was: non stressed control = 8, passive coping = 7, and active coping = 6.

### Experiments 2 and 3: Extinction of contextual fear and c-Fos analyses

The contextual fear paradigm used was previously published^19^ and the experimental design is presented in Fig. 2A–C. The training criterion was that rats display high levels of freezing when re-exposed to the context in which shocks were administered to ensure that large proportions of rats maintained the expression of fear prior to fear extinction retention trials. The contextual fear protocol was as follows: each rat was individually placed in a sound attenuated contextual fear chamber placed in an isolated room (Harvard Apparatus). The floor of each chamber contained stainless steel rods connected to a shock source and grid scrambler that delivered foot shocks as the unconditioned stimulus (US). The chamber contained a low-intensity light and fan, which provided low level background noise. 1% acetic acid was added to the shock grid floor as an olfactory cue. The chamber was cleaned thoroughly between each test subject. Freezing behavior was scored automatically by Packwin software provided with the system and was defined as the absence of movement, except that necessary for breathing, for greater than 2 s, and quantified as percentage of total time for each session.

Contextual fear training occurred 24 h following the 7th and final day of social defeat. Rats were individually placed in the conditioning chamber. The training protocol began with a 210 s period in which subjects were allowed to explore the chamber. Beginning at 211 s, they received five unsignaled footshocks at 1.0 mA, 1 s each, with a 60 s inter-trial interval (ITI). Rats remained in the chamber for 60 s following the last foot shock (pre-end period). On day 9, rats were placed in the same chamber they were trained in for an extinction trial that lasted 8 min. Our criterion for successful learning as assessed during this first extinction trial was that all rats display high levels of freezing during extinction training. The extinction context matched the context rats were trained in (1% acetic acid, fan on, light on). On day 10, rats were tested for retention of extinction using the same contextual cues during an 8 min exposure to the context. On day 21, rats were tested again for persistence of extinction retention.

Three separate cohorts of animals were used in Experiment 2. For c-fos analysis, animals were rapidly decapitated un-anesthetized, and brains were collected and flash-frozen 60 min after the beginning of the 8 min extinction retention trial on day 10. We chose to assess c-Fos after testing for extinction retention, as the specific question of translational relevance was with respect to circuits underlying retention of extinction impairments. Brains were flash frozen in 2-methylbutane and stored at − 80 °C for use in immunohistochemistry and functional connectivity analyses as described below. The total number of subjects used in contextual fear experiments were: non-stressed control n = 18, passive coping n = 20, active coping, n = 22. Of these, the n’s used for c-Fos analyses from Cohort 3 were non-stressed control = 8, passive coping = 7, and active coping = 9.

### Immunohistochemistry (IHC) and assessment of functional connectivity

Brains were sectioned in 30 μm sections for IHC. The brain regions examined are presented in Fig. 3A-B. For the IHC procedure, brain sections were placed in 4% paraformaldehyde for 45 min then sections incubated with the primary antibody for c-Fos (1:1000, Cell Signaling) and using 3,3’-Diaminobenzidine as a chromogen. To analyze images in ImageJ, two researchers blind to the treatment groups counted the number of c-Fos positive cells within a given region of interest. The density of c-Fos-positive cells was averaged over 2–6 sections per animal. Pearson’s *r* correlation matrix was created using c-Fos profiles between all brain regions. Correlations were converted into Fisher’s *z* scores using the *r* to* z* transformation. This transformation converts* r* values into a normal distribution, which allows statistical testing between correlations, and *z* scores of > 1.96 (*i.e.,* equivalent to *p* = 0.05) was considered statistically significant. This is a well-validated method for assessing functional activity between brain regions, which we have previously used^41–43^, and a previous study showed that functional connectivity measures using ΔFosB correlations overlaps with manganese-enhanced MRI of neuronal activity^44^. We have published previous work using this method^29,42,43^. Radar graphs were created for each brain region for additional visualization.

### Data analysis and statistical analysis

The average defeat latency for each rat over the course of the 7 days was calculated and entered into an R script used to perform a bootstrap cluster analyses on the average defeat latencies (code available at www.github.com/cookpa/socialdefeat) as described in Grafe et al. (2018). Latencies are classified as active coping or passive coping based on the probability of resiliency score generated from this analysis that ranges from 0 to 1.0. Active coping animals have probabilities closer to 1.0 while those classified as passive coping have probabilities closer to 0. For statistical comparisons of two groups, we used the Student’s *t* test and for comparisons of more than two groups, we used an analysis of variance (ANOVA). For contextual fear and ethological fear experiments we used repeated measures ANOVAs with time split into 1 min bins in fear testing as the repeated measure. Significant main and interaction effects were followed by post-hoc tests. An α level of 0.05 (two-tailed) was set for significance. Additional analyses were conducted utilizing one-way ANOVAs on the total percent time freezing over the 8 min training sessions. All statistical analyses were made in SPSS version 17, R, or Prism 8. Rats that did not show a conditioned freezing response at greater than 30% freezing at the first two bins of the first fear extinction session were excluded in analyses. Additionally, any values ±2 standard deviations from a group mean were removed from analyses. Based on these criteria, three animals were removed, and the total Ns are reflected above. All data are represented as means ±SEM.

## Dedication

This manuscript is dedicated to the memory of our colleague and friend Leszek Kubin.

## Data Availability

All data generated or analyzed during this study are included in this article. Further enquiries can be directed to the corresponding author [SB].
